# Understanding the Deterioration of Gait, Postural Control, Lower Limb Strength and Perceived Fatigue Across the Disability Spectrum of People with Multiple Sclerosis

**DOI:** 10.3390/jcm9051385

**Published:** 2020-05-08

**Authors:** Pedro Moreno-Navarro, Ramón Gomez-Illán, Carmen Carpena-Juan, Ángel P. Sempere, Francisco J. Vera-Garcia, David Barbado

**Affiliations:** 1 Department of Sports Science, Miguel Hernández University of Elche, 03202 Elche, Spain; pedro.moreno02@goumh.umh.es (P.M.-N.); rjgi78@gmail.com (R.G.-I.); maria.carpena@goumh.umh.es (C.C.-J.); fvera@umh.es (F.J.V.-G.); 2 Department of Clinical Medicine, Miguel Hernández University of Elche, 03550 San Juan de Alicante, Spain; angel.perezs@umh.es; 3 Department of Neurology, University General Hospital of Alicante, 03010 Alicante, Spain

**Keywords:** neurodegeneration, gait, balance, strength, perceived fatigue, relative weight analysis

## Abstract

Disability progression is a prominent feature of multiple sclerosis (MS). However, little is known about the extent to which physical condition parameters and perceived fatigue evolve during the disease. We analyzed how strength, balance, core stability and perceived fatigue differ among different cohorts of people with MS (PwMS) with different disability degrees and how these contribute to patients’ gait speed and functional mobility. Sixty-three PwMS divided into three groups according to the “Expanded Disability Status Scale” (MS1: EDSS ≤ 1.5; MS2: 2 ≤ EDSS ≤ 3.5; MS3: 4 ≤ EDSS ≤ 6) and 22 healthy controls (HC) participated in this study. MS1 showed lower balance and hip strength compared to HC. MS2 showed lower balance, core stability, gait speed, and functional mobility than MS1. MS3 showed lower gait speed, functional mobility, balance, and knee flexion strength than MS2. No between-group differences were observed in perceived fatigue. Relative weight analysis showed that strength, balance and core stability explained 60%–70% of the variance in gait speed and functional mobility. The decline of each parameter did not evolve at the same rate across the different stages of the disease, being knee flexion strength and balance the most influential factors in the disability progression. Overall, these results provide useful information to guide exercise prescription at different stages of MS.

## 1. Introduction

Multiple sclerosis (MS) is a neurodegenerative disease of the central nervous system characterized by a multifocal inflammation, demyelination, reactive gliosis, oligodendrocyte loss, and axonal degeneration [1]. The neurodegeneration caused by the disease entails several motor and cognitive deficits that lead to a progressive disability and have a profound impact on the quality of life of people with MS (PwMS) [2]. In this regard, approximately 80% of MS patients will experience some degree of impairment mobility, which will curtail their ability to support themselves, both financially and physically [3]. The MS treatment entails a considerable socio-economic burden caused by direct and indirect factors [4]. The cost of MS has been estimated at $2.2 million over a person’s lifetime [5]. Among the symptoms caused by the disease, balance and core stability disturbances [6], muscular weakness mainly in the lower limbs [7], and fatigue [8] stand out because they seem to play a prominent role in patients’ loss of functionality [9,10,11,12]. Based on this rationale, patient management programs should aim at improving those deficits that have the greatest impact on the patients’ disability progression. However, the extent to which each of these symptoms should be prioritized to optimize the rehabilitation process is still not clear.

An essential element to take into account when giving priority to improve some deficits over others during rehabilitation programs is to understand how physical condition and fatigue parameters decline throughout the disease process. Some cross-sectional and longitudinal works have shown that disability caused by MS progresses in a non-linear way [13], which suggests that the worsening in the parameters mentioned above may not occur at the same time nor with the same magnitude [14]. However, disability progression in MS is commonly quantified by an increase in the Expanded Disability Status Scale (EDSS), and little is known about the extent to which each specific deficit evolves during the course of the disease relative to the other. Therefore, as a first step, analyzing how all these parameters differ among different cohorts of PwMS could provide useful information to optimize rehabilitation programs according to the stage of the disease of each patient. Additionally, the physical characterization of different cohorts of PwMS could also be used as a hallmark to facilitate the identification of subtle deteriorations caused by the progression of the disease, which, in turn, could be used as indicators for treatment change [15].

Another element to improve our understanding of which deficits must be given priority in a rehabilitation program is to analyze their relative contribution to specific parameters related to the degree of disability. For example, PwMS perceive gait [16] and mobility impairments [17] as two of the most disabling disease symptoms because they hinder their capacity to perform daily living activities (e.g., walking, rising from a chair, turning movements, stair climbing, etc.). In fact, the EDSS is an index of MS disability progression that is heavily determined by ambulation [18]. Several correlational studies have shown that the decline of some factors as the lower limb strength, balance [19], and core stability [10] is highly related to the deterioration of gait speed and functional mobility in PwMS. However, the precise contribution of each of these factors on gait and functional deterioration is still not known. Most studies have used bivariate correlational analyses, which only provide single and unadjusted information about each factor effect on the criterion. In addition, although some works have used the multiple regression analysis to identify the pooled effect of the strength and balance on gait speed [19,20], this analysis shows some limitations in quantifying the relative contribution in terms of explained variance when the factors are correlated [21]. This problem can be especially relevant in MS because the deteriorations in physical factors caused by the disease are usually associated between each other [8,22,23]. To face this issue, Yang et al. [22] have recently proposed the use of relative weight analysis (RWA). This tool reduces the effect of the multicollinearity calculating the proportional contribution of each factor to the criterion variance by adding up both its direct contribution and its combined contribution with other correlated factors [24]. To the best of the authors knowledge, only one study [22] has applied RWA to analyze the degree to which different predictors contributed to gait speed in PwMS. However, in that study, the potential influence of other relevant parameters as core stability, hip abduction/adduction strength, and perceived fatigue on gait speed or functional mobility were not taken into account.

The main aim of this study was to assess differences in lower limb strength, balance, core stability and perceived fatigue between ambulatory PwMS with different disease severity and healthy controls (HC). Specifically, three groups of ambulatory PwMS who presented a range of disease severity were compared: minimal (MS1: EDSS ≤ 1.5), mild (MS2: 2 ≤ EDSS ≤ 3.5) and moderate disability (MS3: 4 ≤ EDSS ≤ 6). The second aim was to examine the relative contribution of these parameters on gait speed and functional mobility using RWA.

## 2. Experimental Section

### 2.1. Participants

Sixty-three participants with MS were recruited from regional patient associations. Additionally, 22 HC were recruited from the patients’ sex/age-matched friends and relatives. PwMS met the following inclusion criteria: (1) diagnosis of MS according to the 2017 revision of the McDonald criteria [25]; (2) they had to be free of relapse in the 90 days prior to testing; (3) age ranging had to be from 18 to 59 years; (4) EDSS ≤ 6; (5) they had to be able to walk 100 m with or without an assistive device; (6) they had to have been undergoing a stable disease-modifying therapy (DMT) regimen for at least 6 months prior to testing. Additionally, participants were excluded if: (1) they needed an orthosis for stance control of the foot, ankle and/or knee; (2) they had any contraindication to perform resistance exercises or (3) they suffered a disability caused by any other medical condition different from MS.

Information regarding demographic characteristics, EDSS, MS type and relevant treatments is summarized in Table 1. The most common MS clinical type was relapsing-remitting (86%), followed by secondary progressive (11%) and primary progressive (3%). Most patients were being treated with DMTs (87.3%). Only 6 patients (9.5%) had been prescribed fampridine to improve gait. No patient presented significant cognitive deficits according to the neurological evaluation (author: AP) although no formal neuropsychological tests were performed.

Based on previous studies’ classifications, participants were stratified into three groups after their recruitment according to their EDSS assessed by the patient’s neurologist [26,27]: those with minimal (MS1: EDSS ≤ 1.5), mild (MS2: 2 ≤ EDSS ≤ 3.5) and moderate disability (MS3: 4 ≤ EDSS ≤ 6). Demographic and clinical descriptive data of each group were obtained from their medical records (Table 1). The experimental procedure was approved by the Local Research Ethics Committee (DPS.RRV.02.14) following the Declaration of Helsinki. All participants provided written informed consent before the data collection.

### 2.2. Experimental Procedures

The physical assessment session was carried out in a biomechanics laboratory with a temperature ranging between 20 and 25 °C. All participants were asked to refrain from doing exercise 48 h prior to testing to reduce the potential influence of fatigue on the physical tests.

During the testing session, MS participants filled out the Dizziness Handicap Inventory (DHI) and the Modified Fatigue Impact Scale (MFIS) questionnaires to assess their self-perception of balance [28] and fatigue [29], respectively. Secondly, participants carried out two tests to assess whole-body balance and core stability: the Tandem Stance Balance test (TS) [10] and the Unstable Sitting Balance test (US) [10]. Subsequently, they performed the Timed 25-Foot Walk test (T25FW) [30] and the Timed Up & Go test (TUG) [31] to assess gait speed and functional mobility, respectively. After that, participants carried out an isokinetic and an isometric dynamometric protocol to assess knee and hip strength, respectively. Finally, participants performed the 6-min walk test (6MWT) [32] to assess their walking endurance.

#### 2.2.1. Balance and Core Stability Test

Participants’ balance and core stability were assessed in tandem and sitting stance, respectively, following the protocol developed by Barbado et al. [10]. Specifically, the TS evaluated the dynamic control of the participants’ body through an anterior-posterior tracking task while standing in tandem position on a force platform (9286AA, Kistler, Winterthur, Switzerland). Tracking movement was carried out using visual feedback in real-time of the participants’ center of pressure (CoP; sampling frequency: 1000 Hz) and of a target point, which repeatedly moved over an anterior-posterior trajectory, taking 20 s to complete a cycle (0.05 Hz). A 2° inclination angle of the whole-body center of mass was calculated to determine the movement amplitude of the target point. The body center of mass was set at 55% of each participants’ height [33]. Before each trial, the initial position of the target point was set by averaging the CoP position during a 6 s static data collection without visual feedback. Participants carried out three 70 s trials with their right leg placed behind their left leg and vice versa, with 1 min rest between trials. The leg order was randomized between participants. In order to standardize practice trials between participants, no familiarization trials were allowed. During each trial, a researcher was by the side of the participants to prevent participants from suffering a possible fall.

After the TS, participants carried out the US to assess participants’ core stability. US was performed on an unstable wooden chair placed on a force platform (sampling at 1000 Hz) [10] to assess core stability. A polyester-resin hemisphere (diameter = 35 cm; height = 12 cm) was attached to the chair bottom. Participants were seated with their arms crossed over their chest and their legs strapped to the chair at 90° of knee flexion to avoid lower limb influence on postural control. In the same way as TS, the feedback of the CoP and the target point was provided in real-time. Nevertheless, in this test, the target point moved over a circular trajectory (20 s to complete a cycle; 0.05 Hz). A 4° inclination angle of the upper-body center of mass was calculated to determine the movement amplitude of the target point. The center of mas of the upper body was set at 62.6% of the distance between the greater trochanter and the acromioclavicular joint [33]. Similar to the TS, the target point position was readjusted before each trial by averaging the CoP position during a 6 s static data collection without visual feedback. Participants performed five 70 s trials with 1 min rest between trials. No familiarization trials were allowed in order to standardize practice trials between participants. Although a support rail placed around participants was provided to prevent them from falling, all participants were able to maintain the sitting position without having to hold on to it in at least 3 of the 5 trials.

#### 2.2.2. Gait Speed and Functional Mobility Tests

After the postural control tests, participants performed the T25FW [30] and the TUG [31] to assess gait speed and functional mobility, respectively. Unlike most previous versions of these protocols, in order to induce higher neuromuscular demands to reduce outcome variability (which helps to improve test reliability), participants were requested to carry out both tests in the shortest time possible [10]. Therefore, they were allowed to run if they could. Specifically, in the T25FW, participants had to cover a distance of 25 feet (7.62 m). Similarly, in the TUG, participants had to stand up from a chair, move forward 3 m, turn around a cone and sit back down on the chair. In both tests, time (s) was recorded using a digital chronometer (HS-30W-N1V, CASIO, Tokio, Japan). Participants performed three trials of the TUG and the T25FW with 1 min rest between trials.

#### 2.2.3. Lower Limb Strength Tests

Knee muscle strength was evaluated using the Biodex System-4 isokinetic dynamometer (Biodex System-4, Biodex Corp., Shirley, NY, USA). Participants were seated and strapped at their chest, hip, thigh and foot to obtain an isolated measure of quadriceps and hamstring strength. The attachments of the dynamometer were adjusted so that the rotation axis of the lever arm was aligned with the lateral epicondyle of the knee [34]. The range of motion was set within 10°–90° for the knee joint considering the straight leg as 0°. Participants carried out three series of repetitions of isokinetic (concentric/concentric) knee extension and flexion at 60°/s with 2 min rest between series. Participants were verbally encouraged to perform their best during the protocol. Before the isokinetic testing, participants warmed up by performing two series of 10 sub-maximal repetitions (self-perceived 50% effort) at the same isokinetic speed. The leg order was randomized between participants. Each participant’s best trial from each leg (i.e., maximum peak torque) was normalized by her/his body mass (N×m^2^/kg) and used for further statistical analyses.

Additionally, isometric hip abduction and adduction were assessed using a portable handheld dynamometer (Manual Muscle Tester; Lafayette Indiana Instruments, Lafayette, IN, USA) according to the protocol described by Mañago et al. [35]. During the test, participants lied in a supine position, with their legs extended on a stretcher, stabilizing themselves with their hands grasped to the stretcher sides. Specifically, participants performed two practical trials (50% and 80% of the self-perceived maximal voluntary contraction) and three 5 s isometric maximal voluntary contraction trials for each hip movement direction of each leg. During each maximal voluntary contraction, participants were requested to push against the dynamometer held by a researcher, increasing force during the first 3 s and trying to perform their maximum for the last 2 s. The hand-held dynamometer was placed at 5 cm proximal to the lateral and medial malleolus for the abduction and adduction efforts, respectively. The order of the leg and the abduction/adduction exertion were randomized between participants. Each participant’s best trial from each leg (i.e., maximum peak force) was normalized by the body mass (N/kg) and used for further statistical analyses.

#### 2.2.4. Walking Endurance

To analyze the walking endurance, participants performed the 6MWT [32]. Participants were instructed to walk as far as possible for 6 min on a rectangular track (20 × 10 m), encouraging them to keep a constant pace. Nevertheless, they were permitted to slow down if necessary. Participants were not allowed to use any assistive device to be able to analyze the true contribution of each participant’s balance to gait performance. A researcher walked alongside each participant along the track to avoid the possibility of any fall.

### 2.3. Data Reduction

CoP signal recorded during the TS and the US was low-pass filtered (4th-order, 0-phase-lag, Butterworth, 5 Hz cut-off frequency) [36] and subsequently subsampled at 20 Hz. The first 10 s of each trial were also removed to reduce the potential influence of the non-stationary behavior related to the beginning of the trial [37]. Subsequently, participants’ postural control while standing and sitting was quantified by calculating the mean radial error (i.e., the average of the CoP vector distance magnitude from the target point).

Finally, as the weaker leg muscle strength seems to be more related to walking performance than the stronger leg in PwMS [9], participants’ legs were categorized as stronger or weaker leg based on the torque peak obtained during the isokinetic test. Therefore, we analyzed eight strength parameters in this study: (a) isokinetic knee extension peak torque for the weakest leg (EXT_WL_) and for the strongest leg (EXT_SL_), (b) isokinetic knee flexion peak torque for the weakest leg (FLX_WL_) and for the strongest leg (FLX_SL_), (c) isometric hip adduction peak force for the weakest leg (ADD_WL_) and for the strongest leg (ADD_SL_), (d) isometric hip abduction peak force for the weakest leg (ABD_WL_) and for the strongest leg (ABD_SL_). Moreover, TS outcomes were categorized as tandem stance performance with the stronger leg placed behind the weaker leg (TS_SL_) or tandem stance performance with the weaker leg placed behind the stronger leg (TS_WL_).

### 2.4. Statistical Analysis

Descriptive statistics (mean ± SD) from each parameter were calculated for every group. The normality of the data was assessed using a Kolmogorov–Smirnov test with the Lilliefors’ correction. One-way independent-measures ANOVAs were performed to assess between-group differences for each parameter, being group the between-subject factor [4 levels: the control group and the minimally (EDSS ≤ 1.5), mildly (2 ≤ EDSS ≤ 3.5) or moderately impaired (4 ≤ EDSS ≤ 6) PwMS groups]. Although participants’ *age* was not homogenous across groups, no ANCOVAs were carried using age as a covariate because the homogeneity of the regression slope assumption was not met. Post hoc analysis with Tukey or Games–Howell adjustments were used for multiple comparisons when the assumption of homogeneity of variances was or was not accomplished, respectively. Hedges’ *g* index (*d_g_*) was used to estimate the effect size of each pairwise comparison [38]. This index is based on Cohen’s *d* index [39], but it provides an effect size estimation reducing the bias caused by small samples (*n* < 20). According to Cohen [39], effect sizes were categorized as: trivial (*d_g_* < 0.2), small (0.2 ≤ *d_g_* <0.5), moderate (0.5 ≤ *d_g_* < 0.8) and large (*d_g_* ≥ 0.8). ANOVAs were performed with the SPSS (Version 22.0, IBM, Armonk, NY, USA), establishing significance at *p* < 0.05. Additionally, the percentage differences (PD) in the different parameters between the three MS groups compared to the HC were calculated in order to provide a more clinically meaningful information about the deterioration caused by the MS disease. For Hedges’ *g* and PD, positive scores indicate a deterioration of the group with higher EDSS scores compared to the group with lower EDSS scores or with the control group. Finally, RWA [24] was used to examine the relative contribution of each parameter in explaining the variance in the TUG and T25FW performance using the RWA Web [40]. All potential factors meeting the assumptions of normality and homoscedasticity were entered into the RWA. A backward elimination procedure was used to remove all those parameters that did not influence the TUG and the T25FW (*p* > 0.05) significantly. The relative importance of each factor was calculated as the percentage of the T25FW or TUG variance (*R*^2^) that they explained. The relative importance of each factor was also compared with the other factors.

Before performing the ANOVAs and RWAs, the sampling software package GPower 3.1. [41] was used to calculate the minimum sample size needed to detect significant results in each statistical analysis. A sample size of 19 participants per group was found to be necessary to detect large significant main effects in ANOVAs (*F* = 0.4; power = 80%; α = 0.05). Taking into account the high heterogeneity shown by PwMS (i.e., large group variance) as well as the high within-subject variability [42,43], large effect sizes must be observed to find statistically significant between-group differences. Therefore, for pairwise between-group comparisons, a sample size of 21 participants per group was needed to detect large differences between each group (*d_g_* = 0.8; power = 80%; α = 0.05). Finally, regarding the RWA, based on previous results [22], a sample of 61 participants was needed to detect a significant large effect size (*f*^2^ = 0.35; power = 80%; α = 0.05) on a multiple linear regression model with 12 potential predictors. According to this sample size estimation explained below, RWA was applied in the whole sample of PwMS.

## 3. Results

All participants completed all the tests except for four participants from the MS3 group who were not able to accomplish the TS_WL_, and two of them were not able to accomplish the TS_SL_ either. All the tests were well-tolerated and no injuries, exacerbations and/or relapses happened during the study.

### 3.1. Differences between Disability Stages

The comparison between the HC and the MS groups can be seen in Table 2. Overall, MS groups showed lower performance than the HC in all physical condition parameters. Although the physical deterioration was significantly higher with the advance of the disease (*p* < 0.001), the decline of each parameter did not evolve at the same magnitude across the different stages of the disease (i.e., minimal, mild and moderate impairment). The MS2 and MS3 groups showed significant differences in all physical parameters compared to HC. However, when the MS1 and the HC were compared, the highest differences were found in TS (0.94 ≤ *d_g_* ≤ 1.18), in ABD_SL_ and ABD_WL_ (1.04 ≤ *d_g_* ≤ 1.18) and in ADD_WL_ (*d_g_* = 0.82). No significant differences were observed for the gait and functional test, nor for knee strength. When the MS1 and MS2 groups were compared, MS2 participants showed significant reductions and the largest effect sizes in TS (0.96 ≤ *d_g_* ≤ 1.07), US (*d_g_* = 0.96), TUG (*d_g_* = 0.91), T25FW (*d_g_* = 1.19) and 6MWT (*d_g_* = 1.05). No significant differences were observed for hip strength, DHI and MFIS. Lastly, MS3 group showed significant differences in all physical parameters compared to MS1. Regarding the MS2 and MS3 group comparison, MS3 participants presented significant reductions and the highest effect sizes in TS (1.31 ≤ *d_g_* ≤ 1.64), US (*d_g_* = 1.00), TUG (*d_g_* = 2.34), T25FW (*d_g_* = 2.61), 6MWT (*d_g_* = 1.78) and in FLX_SL_ and FLX_WL_ (1.24 ≤ *d_g_* ≤ 0.97). DHI also showed significant differences between groups (*d_g_* ≤ 0.75). No significant differences were observed between groups for knee extensor strength, hip strength, and MFIS.

PD used as clinical indexes of deterioration (Table 2) qualitatively confirmed that the decline of the physical parameters caused by MS did not evolve in the same degree. MS1 showed poor balance (TS: 25.9% ≤ PD ≤ 37.5%) and lower hip strength (24.7% ≤ PD ≤ 29.9%) than HC. When MS2 was compared to HC, TS and US, it showed the largest between-group differences (76.9% ≤ PD ≤ 88.8%). Finally, MS3 showed the biggest differences in TS and US (183.2% ≤ PD ≤ 226.3%), in the TUG and T25FW (119.3% ≤ PD ≤ 156.3%) and in FLX_SL_ and FLX_WL_ (39.3% ≤ PD ≤ 58.9%) compared to HC.

### 3.2. Relative Weight Analysis

As four participants of the MS3 were not able to perform the TS_WL_, the RWA were carried out using data from the 59 remaining PwMS. The RWA results showed that strength, balance and core stability parameters explained a high variance in the 6MWT (71.0%), T25FW (60.5%) and TUG (61.2%) performance, respectively (Table 3). Out of the nine factors significantly associated with the 6MWT, all strength parameters explained 51.4% of the 6MWT performance variance. Among them, the knee flexion strength showed a higher contribution to the variation in the 6MWT performance (FLX_WL_ = 16.4%; FLX_SL_ = 14.8%) than the knee extensor strength (EXT_WL_ = 6.4%; EXT_SL_ = 4.5%) and the hip abduction strength (ABD_SL_ = 4.9%; ABD_WL_ = 4.3%). TS_WL_ and FLX_WL_ were the most important determinants to explain the variance in the T25FW performance (FLX_WL_ = 18.8%; TS_WL_ = 18.2%) and the TUG performance (FLX_WL_ = 16.8%; TS_WL_ = 13.3%). FLX_SL_ was revealed as another important factor for both the T25FW (11.6%) and the TUG (9.4%) performance variance. Core stability was more determinant for explaining variations in the TUG performance (11.3%) than in the T25FW performance (7.4%). The DHI significantly contributed to explaining the variations in the TUG performance (5.2%) but not the T25FW performance. EXT_WL_ had a smaller weight in explaining the T25FW (4.6%) and the TUG (5.7%) performance variance. The RWA results also pointed out that the weight of the EXT_WL_ to predict T25FW or TUG variance was significantly lower than TS_WL_ or FLX_WL_. MFIS did not show a significant result in any RWA model. No parameter showed a significant higher weight than others to predict 6MWT variance.

## 4. Discussion

The main aim of this study was to analyze, through the comparisons of different cohorts of PwMS, how lower limb strength, balance, core stability and perceived fatigue decline because of the MS progress, and how these factors are associated to functional mobility and gait speed. The main findings were that the strength, balance and core stability deterioration observed across the MS groups seems to be different between physical parameters unlike perceived fatigue that shows no change between MS groups. RWA models explained about 60% of the variance in gait speed and functional mobility, being knee flexion strength and balance the most influential factors.

### 4.1. The Decline of Physical Parameters in the Early Stages of the Disease (HC vs. MS1)

The main point of attention regarding the comparison between the PwMS in the early stages of the disease (EDSS ≤ 1.5; patients with minimal or non-disability according to the neurological evaluation) and participants without the disease was that, although MS1 seems to show slightly worse performance in most of the parameters (Table 2), significant differences were only found for balance (0.94 ≤ *d_g_* ≤ 1.18; 25.9% ≤ PD ≤ 37.5%) and hip strength (0.73 ≤ *d_g_* ≤ 1.18; 24.7% ≤ PD ≤ 29.9%), mainly in the abductor muscles. These results seem to confirm previous findings in which posturographic tests revealed subtle balance deficits in the early stages of the MS disease in the absence of clinical disability in gait and functional tasks [44]. Interestingly, the lack of significant differences in core stability supports the idea that before this parameter is affected, balance deterioration can be observed in those tasks involving lower limb participation [10]. Together with this balance deterioration, a reduction of hip strength was observed; however, this was not observed in the knee muscles (5.7% ≤ PD ≤ 12.4%). To the best of the authors’ knowledge, the rationale supporting these results is unclear. A potential explanation could be related to the sedentary behavior of PwMS [45] that could lead to further deconditioning of those muscles which are not challenged during daily life activities as knee muscles are (e.g., walking, stair climbing, etc.).

Our results highlight the importance of performing balance and hip strength exercises during the early stage of the MS, even when there is no clear evidence of clinical disability. Interestingly, they also suggest that at this stage of the disease, gait and functional test such as the 6MWT, T25FW or TUG are not useful tools to assess the early disability caused by MS. Conversely, the clinical evaluation of PwMS in these stages of the disease could be focused on revealing subtle balance and hip strength deficits. Therefore, considering the high cost and relatively complex data reduction when using force platforms, the development of clinical tests that can be applied in professional settings while keeping comparable assessment accuracy to laboratory-based protocols would be needed. For example, future studies should evaluate the suitability and reliability of using wearable technologies (e.g., accelerometers embedded into smartphones) [46,47] to assess balance and core stability in PwMS.

### 4.2. The Decline of Physical Parameters in Mildly Impaired PwMS (MS1 vs. MS2)

The first point of interest is that mild impaired PwMS (MS2: 2≤EDSS≤3.5) showed a significant physical deterioration for all parameters compared to HC. The gait speed and functional mobility of the MS2 group were also impaired (0.91 ≤ *dg* ≤ 1.19) when compared with minimally impaired (MS1) PwMS. This functional deterioration was accompanied by a significant decline in balance and core stability (0.96 ≤ *dg* ≤ 1.07) compared to MS1, reaching PD that ranged from 76.9 to 88.8% compared to HC. Interestingly, the DHI was not able to detect balance differences between groups, confirming that posturography is a more sensitive tool to assess balance impairments [6,28]. These results also support the need for introducing wearable technologies in clinical assessments to obtain a sensitive balance evaluation comparable to laboratory equipment [48]. Although most of the knee and hip strength tests did not show significant differences between MS1 and MS2, a high strength reduction was observed based on the effect size index (0.49 ≤ *d_g_* ≤ 0.81). Both groups showed a reduction in lower limb strength that ranged from 20 to 40% compared to HC (Table 2). It should be noted that PwMS in MS2 were significantly older than PwMS in MS1, so inter-group strength and balance differences could not be totally attributed to the advance of the disease.

In short, these results confirm the strength and balance reduction in mildly impaired PwMS observed in previous studies [7,49]. This overall physical decline seems to be the cause of the worse performance by MS2 in the 6MWT, TUG and T25FW compared to the minimally impaired PwMS (MS1). Based on these results, as the restoration of the physical capacities might be more challenging than their preservation, from the authors’ point of view, preventive and broad training programs working all physical capabilities should be implemented even before PwMS reaches these stages of the disease.

### 4.3. The Decline of Physical Parameters in Moderately Impaired PwMS (MS2 vs. MS3)

Regarding the most disabled ambulant PwMS (MS3), they showed a significant physical deterioration for all parameters compared to MS1, although these differences could be not totally attributed to the advance of the disease because PwMS in MS3 were slightly older than PwMS in MS1. Comparing MS3 with MS2, gait speed and functional mobility showed a huge deterioration (1.78 ≤ *d_g_* ≤ 2.61). The PwMS at this stage of the disease took more than twice as long to finish both the T25FW and the TUG compared to HC. They also walked close to three times less distance in the 6MWT, which confirms EDSS scores of 4 or higher as a specific disability point in which gait and related tasks are exponentially deteriorated [50]. This exponential deterioration of gait speed and functional mobility could be related to the enormous decline of balance (1.31 ≤ *d_g_* ≤ 1.64), core stability (*d_g_* = 1.00) and knee flexor strength (0.97 ≤ dg ≤ 1.24), mainly observed in the weaker leg. In this sense, the hamstring weakness observed in the MS3 may be related to the lower knee flexion during the swing phase of the gait commonly observed in PwMS [51]. In this stage, DHI was able to detect balance differences between groups, but the effect size was smaller (*d_g_* = 0.75) than those shown by posturographic tests, confirming the lower sensitivity of DHI to detect subtle changes [49]. Interestingly, no significant reduction in knee extensor and hip strength was found when comparing MS2 and MS3, suggesting that they should not be the main target of rehabilitation programs at this stage of the disease.

Based on these results, the high degree of impairment of the MS3 highlights the need for intensive rehabilitation programs to improve all physical abilities and ultimately to preserve (or restore) functionality of PwMS with EDSS ≥ 4. Besides, the high balance and core stability impairments observed in MS3 seem to indicate that these qualities should be prioritized in rehabilitation programs. In addition, resistance training programs for PwMS at this stage of the disease should also focus on flexor strength, especially in the weaker leg, as hamstrings showed a strength reduction of 58.9% compared to healthy counterpart people.

### 4.4. Perceived Fatigue across the Different Cohorts of PwMS

Perceived fatigue is reported by patients as one of the most disabling and frequent symptoms that affect their quality of life [52]. In this sense, several cross-sectional works have identified that higher levels of perceived fatigue are associated with higher disability and advanced stages of the disease (i.e., EDSS, age since MS was detected, etc.) [52,53]. However, contrary to what was expected, we did not find any significant differences in the MFIS between any MS groups. Our results are in line with other studies that failed to find any correlation between perceived fatigue and different indexes of the advance of the disease and disability such as EDSS scores [54] or global white matter damage [55,56]. Overall, these results suggest that the disabling effect of the perceived fatigue on PwMS might not have a prominent link to the physical impairment caused by the disease.

### 4.5. Relative Weight Analyses

The RWA model confirmed the relevance of lower limb strength for the gait speed and functional mobility, as it accounted for about 60% of the variation in the T25FW and TUG performance and for 71% of variation in the 6MWT performance. Interestingly, the amount of explained variance by all strength and balance parameters was similar than that observed by Fritz et al. [57] but higher than that observed in previous studies [19,22], in which both parameters explained from 30 to 40% of the gait speed variance. From the authors’ point of view, these between-study differences seem to be more related to the impact of the balance-related parameters rather than the strength ones. Firstly, similar to Fritz et al. [57], we used a highly demanding postural control task which challenged balance in dynamic conditions, asking participants to track a target displayed on a screen with their COP on a reduced medial-lateral base of support (i.e., tandem stance) [10]. In this sense, more challenging tasks require a tighter neuromuscular control, which allows to reveal the true balance performance of an individual [58], which, in turn, would help to identify the real impact of balance on gait or functional mobility. Secondly, in our study, we introduced core stability as a novel predictor that showed a significant influence on gait speed (7.4%) but especially on functional mobility (11.3%). These results emphasize the incremental role that core stability plays in accomplishing gait-related functional tasks that include such demanding actions as rising from a chair or turning [10]. Although more studies are needed to confirm the impact of this factor on PwMS’ mobility, our results reinforce the idea of introducing core stability exercises in rehabilitation programs to improve PwMS’ ability to perform daily living activities. Finally, DHI was observed to be a small but significant predictor for functional mobility (TUG: 5.2%) and the walking endurance (6MWT: 4.9%), supporting the idea that a poor balance self-perception contributes to reducing the PwMS’ functional mobility. This phenomenon would be closely related to the significant impact that fear of falling has on the reduction of the functional capacity in PwMS [6].

Regarding the strength parameters, their total impact on the T25FW (35.8%) and TUG (33.2%) performance was similar to that observed by Yang et al. [22], who found that this factor explained the 32.1% of the gait speed variance. Remarkably, the contribution of strength parameters was higher for the 6MWT performance (51.4%) than for the T25FW, highlighting the relevance of this factor to perform prolonged physical activities [59]. Importantly, our design allowed us to identify the relative contribution of several muscle groups. Among the strength parameters, our results confirmed previous findings [9] showing that the deteriorations in maximal walking distance, gait speed, and functional mobility are mainly attributed to the knee flexor muscles (9.4% ≤ R^2^ ≤ 18.8%), especially to the status of the weaker leg while knee extensor and hip muscles showed a small contribution (<6.5%) [9]. These results emphasize the need for strengthening programs to prioritize the improvement of the weaker leg with particular attention to the hamstring muscles [60].

Regarding perceived fatigue, the fact that MFIS scores did not show a significant impact on the 6MWT, T25FW and TUG performance questions the potential influence of the perceived fatigue on PwMS’ functional capacity. It must be noted that perceived fatigue was linked with gait speed in prolonged walking tasks [61]; however, we did not find similar results in our study. Future studies are needed to determine the precise role of physiological fatigue and perceived fatigue caused by the disease on the functional capacity of PwMS.

In summary, based on between-groups comparisons and RWA, the decline of each parameter did not evolve at the same magnitude across the different stages of the disease, and these parameters did not have the same impact on gait and functional mobility. Therefore, exercise interventions should focus on different capabilities according to the MS stage in which each patient is and help to implement preventive exercise programs aimed to reduce the expected decline in physical capacity in future stages of the disease. In Table 4, we have provided some general guidelines about what exercises or capabilities must be given priority in a rehabilitation program according to the EDSS stage of each MS patient.

### 4.6. Limitations

Besides, although the sample could be considered adequate for biomechanical studies, it is still too small to provide normative scores to categorize different cohorts of PwMS and to control the potential bias that confounding parameters (e.g., age, years with the disease, lifestyle, etc.) could have had on our results. In addition, based on the sample size limitations, our RWA results should be interpreted with caution, focusing on determining which group of factors are more important than others rather than on establishing a specific rank ordering [62]. Moreover, although the patients’ treatment was not modified in the six months prior to the testing, some patients in the MS3 group received 4-Aminopyridine (Table 1), which has been shown to improve the gait speed in PwMS [63]. Thus, although the MS3 group showed significantly worse gait speed and endurance scores than the other groups, gait deficit at the stage of the disease could have been underestimated. Another limitation is the lack of testing of some elements that the literature has shown have an impact on gait speed and endurance results (e.g., spasticity, pain, sensorial impairments, ankle dorsiflexion strength and range of movement [51,64,65]) in RWA in order to explain the 40% of the variance non-accounted for by our model. Finally, it must be highlighted that these findings provide a general exercise guideline according to the MS stage in which each patient is. However, training programs should be individualized according to an exhaustive evaluation of the evolution of each patient’s deficits.

## 5. Conclusions

The results of this cross-sectional study provide clinicians and researchers with a description of the way that physical deterioration may evolve throughout the MS disease, which can help to improve training and rehabilitation programs. In the early stages of the disease (EDSS ≤ 1.5), only balance and hip strength parameters showed a significant reduction which ranged from 24% to 38% compared to HC. In mildly impaired PwMS (2 ≤ EDSS ≤ 3.5), an overall physical decline was observed, and thus, a comprehensive training program should be implemented to preserve or restore the motor function at this stage. Finally, the most disabled ambulant PwMS (4 ≤ EDSS ≤ 6) showed an important deterioration of the balance and hamstring strength of the weakest leg compared to HC with PD of 226.4% and 58.9%, respectively, which reinforces the need to focus training programs on the most disable leg as a crucial factor to increase functionality at this stage of the disease. Core stability plays a relevant role in gait-related tasks that include such demanding actions as rising from a chair or turning. Finally, the levels of perceived fatigue do not seem to be directly linked to the physical deterioration caused by the disease.

## Figures and Tables

**Table 1:** Demographic, clinical characteristics and medications of the healthy controls and multiple sclerosis individuals stratified according to their Expanded Disability Status Scale (EDSS).

	HC	MS1	MS2	MS3	F	*p*
**Age (years)**	40.23 ± 8.15	36.70 ± 7.33	44.96 ± 7.77 *	44.65 ± 8.03 *	5.24	0.002
**Height (m)**	165.34 ± 7.79	167.73 ± 9.68	163.57 ± 7.68	166.25 ± 10.64	0.32	0.812
**Body mass (kg)**	66.99 ± 14.05	67.41 ± 10.07	64.21 ± 11.49	65.84 ± 15.17	0.81	0.494
**EDSS**	-	1.10 ± 0.60	2.83 ± 0.51	5.08 ± 1.00	150.28	<0.001
**Disease Duration (years)**		6.15 ± 4.31	9.96 ± 5.30	11.60 ± 6.91 *	5.02	0.010
**Female/Male (*n*)**	18/4	16/4	20/3	13/7		
**MS Type (*n*)**						
Relapsing remitting	-	20	21	13		
Secondary progressive	-	-	1	6		
Primary progressive	-	-	1	1		
**Disease-modifying drugs (*n*)**						
Alemtuzumab	-	1	2	1		
Azathioprine	-	0	1	0		
Dimethyl-fumarate	-	0	3	1		
Fingolimod	-	1	6	3		
Interferon-beta	-	4	4	2		
Natalizumab	-	7	1	2		
Ocrelizumab	-	0	1	1		
Rituximab	-	0	1	3		
Teriflunomide	-	2	2	0		
Glatiramer acetate	-	2	2	2		
None	-	3	0	5		
**Fampridine (*n*)**	-	0	0	6		

Note. Values are mean scores ± SD or as otherwise indicated. SD: standard deviation; HC: healthy controls; MS1: multiple sclerosis people with minimal or non-impairment (EDSS ≤ 1.5); MS2: multiple sclerosis people with mild impairment (2 ≤ EDSS ≤ 3.5); MS3: multiple sclerosis people with moderate impairment (4 ≤ EDSS ≤ 6). *: significantly different from MS1.

**Table 2:** Comparison of strength, balance, core stability and perceived fatigue parameters between healthy controls (*n* = 22) and multiple sclerosis individuals with minimal (*n* = 20), mild (*n* = 23) and moderate impairment (*n* = 20).

							Effect Size (Hedge’s *g*)			Percentage Differences		
	HC	MS1	MS2	MS3	F	*p*	HC vs. MS1	MS1 vs. MS2	MS2 vs.MS3	HC vs. MS1	HC vs. MS2 ^†^	HC vs. MS3 ^††^
**Walk endurance, gait speed functional mobility**												
**6MWT**	863.4 ± 145.4	746.6 ± 220.1	558.7 ± 125.6	322.3 ± 135.2	45.62	<0.001	0.62	1.05 *	1.78 *	13.5 ± 25.5	35.3 ± 14.5	62.7 ± 15.7
**T25FW**	2.50 ± 0.32	2.70 ± 0.38	3.24 ± 0.50	6.41 ± 1.67	86.74	<0.001	0.55	1.19 *	2.61 *	7.8 ± 15.1	29.6 ± 20.0	156.3 ± 66.7
**TUG**	4.70 ± 0.55	5.10 ± 0.58	5.86 ± 0.97	10.32 ± 2.54	71.09	<0.001	0.69	0.91 *	2.34 *	8.4 ± 12.4	24.5 ± 20.6	119.3 ± 53.9
**Whole-body balance, core stability and dizziness**												
**TS_WL_**	7.94 ± 1.85	10.92 ± 3.03	14.99 ± 4.96	25.91 ± 8.29	46.43	<0.001	1.18 *	0.96 *	1.64 *	37.5 ± 38.1	88.8 ± 62.5	226.4 ± 104.4
**TS_SL_**	7.22 ± 1.74	9.09 ± 2.17	12.77 ± 4.13	20.45 ± 7.30	36.17	<0.001	0.94 *	1.07 *	1.31 *	25.9 ± 30.1	76.9 ± 57.2	183.2 ± 101.1
**US**	8.35 ± 3.22	10.79 ± 3.56	15.45 ± 5.40	23.91 ± 10.75	23.78	<0.001	0.71	0.96 *	1.00 *	29.2 ± 42.6	85.1 ± 64.7	186.5 ± 128.7
**DHI**	-	23.08 ± 20.01	32.09 ± 19.26	47.70 ± 17.20	8.26	0.001	-	0.47	0.75 *	-	-	-
**Knee strength**												
**EXT_WL_**	1.90 ± 0.35	1.74 ± 0.39	1.43 ± 0.38	1.22 ± 0.46	12.41	<0.001	0.43	0.77	0.50	8.7 ± 20.7	24.7 ± 20.1	35.9 ± 24.1
**EXT_SL_**	2.03 ± 0.37	1.92 ± 0.38	1.61 ± 0.37	1.55 ± 0.35	8.54	<0.001	0.30	0.79 *	0.18	5.7 ± 18.8	20.6 ± 18.2	23.8 ± 17.3
**FLX_WL_**	0.91 ± 0.21	0.80 ± 0.21	0.65 ± 0.21	0.37 ± 0.24	24.37	<0.001	0.54	0.69	1.24 *	12.4 ± 22.7	28.3 ± 22.7	58.9 ± 25.8
**FLX_SL_**	1.01 ± 0.21	0.94 ± 0.18	0.80 ± 0.16	0.61 ± 0.22	16.81	<0.001	0.33	0.81	0.97 *	6.6 ± 18.1	20.7 ± 16.1	39.3 ± 21.7
**Hip strength**												
**ADD_WL_**	2.32 ± 0.93	1.67 ± 0.58	1.42 ± 0.38	1.36 ± 0.38	12.25	<0.001	0.82 *	0.49	0.17	28.2 ± 24.9	38.7 ± 16.4	41.4 ± 16.2
**ADD_SL_**	2.62 ± 1.07	1.97 ± 0.57	1.61 ± 0.40	1.59 ± 0.42	11.00	<0.001	0.73	0.74	0.03	24.7 ± 21.7	38.6 ± 15.1	39.2 ± 16.2
**ABD_WL_**	2.70 ± 0.95	1.89 ± 0.48	1.64 ± 0.39	1.39 ± 0.51	17.93	<0.001	1.04 *	0.58	0.53	29.9 ± 17.7	39.4 ± 14.6	48.6 ± 19.0
**ABD_SL_**	3.10 ± 0.94	2.18 ± 0.50	1.81 ± 0.43	1.79 ± 0.52	20.43	<0.001	1.18 *	0.78	0.04	29.7 ± 16.0	41.6 ± 14.0	42.2 ± 16.7
**Perceived fatigue**												
**MFIS**	-	40.55 ± 15.29	44.83 ± 12.01	40.95 ± 14.97	0.620	0.54	-	0.31	−0.28	-	-	-

Note. Values are mean scores ± SD. For Hedges’ g and Percentage differences, positive scores indicate a deterioration of the group with higher EDSS scores compared to the group with lower EDSS scores or with the control group. ^†^ MS2 showed significant pairwise differences in all parameters compared to HC; ^††^ MS3 showed significant pairwise differences in all parameters compared to HC and MS1; * Significant differences between groups at *p* < 0.05 adjusted with the Tukey or Games–Howell corrections when the assumption of homogeneity of variances is or is not accomplished, respectively. SD: standard deviation; HC: healthy controls; MS1: multiple sclerosis people with minimal impairment (EDSS ≤ 1.5); MS2: multiple sclerosis people with mild impairment (2 ≤ EDSS ≤ 3.5); MS3: multiple sclerosis people with moderate impairment (4 ≤ EDSS ≤ 6); EDSS: Expanded Disability Status Scale; 6MWT: 6-min Walk test (m); T25FW: Timed 25-Foot Walk test (s); TUG: Timed Up & Go test (s); TSWL: Tandem Stance Balance test with the weakest leg behind (mm); TSSL: tandem stance balance test with the strongest leg behind (mm); US: Unstable Sitting Balance test (mm); DHI: Dizziness Handicap Inventory (unitless); EXTWL: isokinetic knee extension peak torque for the weakest leg (N×m/kg); EXTSL: isokinetic knee extension peak torque for the strongest leg (N×m/kg); FLXWL: isokinetic knee flexion peak torque for the weakest leg (Nm/kg); FLXSL: isokinetic knee flexion peak torque for the strongest leg (Nm/kg); ADDWL: isometric hip adduction peak force for the weakest leg (N/kg); ADDSL: isometric hip adduction peak force for the strongest leg (N/kg); ABDWL: isometric hip abduction peak force for the weakest leg (N/kg); ABDSL: isometric hip abduction peak force for the strongest leg (N/kg); MFIS: Modified Fatigue Impact Scale (unitless).

**Table 3:** Relative Weight Analysis with the 6MWT, T25FW and TUG as the dependent variables and their significant (*p* < 0.05) predictive factor (PF) in people with multiple sclerosis (*n* = 61).

		Explained Variance (%, Adjusted R^2^)									
	Total		1st PF	2nd PF	3rd PF	4th PF	5th PF	6th PF	7th PF	8th PF	9th PF
**6MWT**	71.0	**Predictor**	FLX_WL_	FLX_SL_	TS_WL_	TS_SL_	EXT_WL_	ABD_SL_	DHI	EXT_SL_	ABD_WL_
		**Mean**	16.4	14.8	7.7	7.0	6.4 ^A^	4.9	4.9	4.5	4.3
		**(95% CI)**	(10.6–22.7)	(9.1–21.7)	(3.6–12.3)	(2.9–13.2)	(2.7–10.9)	(1.4–9.3)	(1.4–9.9)	(1.6–8.4)	(1.3–7.6)
**T25FW**	60.6	**Predictor**	FLX_WL_	TS_WL_	FLX_SL_	US	EXT_WL_				
		**Mean**	18.8	18.2	11.6	7.4	4.6 ^A,B^				
		**(95% CI)**	(7.8–31.3)	(7.9–34.5)	(5.3–20.2)	(1.9–17.4)	(1.5–9.7)				
**TUG**	61.7	**Predictor**	FLX_WL_	TS_WL_	US	FLX_SL_	EXT_WL_	DHI			
		**Mean**	16.8	13.3	11.3	9.4	5.7 ^A^	5.2 ^A^			
		**(95% CI)**	(6.5–28.9)	(4.9–27.7)	(4.1–22.8)	(3.5–14.9)	(1.6–11.4)	(1.2–11.6)			

^A^: significant differences compared to the 1th PF; ^B^: significant differences compared to the 2th PF; CI: Confidence interval; 6MWT: 6-min Walk test (m); T25FW: Timed 25-Foot Walk test (s); TUG: Timed Up & Go test (s); TS_SL_: Tandem Stance Balance test with the strongest leg behind (mm);TS_WL_: Tandem Stance Balance test with the weakest leg behind (mm); US: Unstable Sitting Balance test (mm); EXT_WL_: isokinetic knee extension peak torque for the weakest leg (Nm/kg); EXT_SL_: isokinetic knee extension peak torque for the strongest leg (Nm/kg); FLX_WL_: isokinetic knee flexion peak torque for the weakest leg (Nm/kg); FLX_SL_: isokinetic knee flexion peak torque for the strongest leg (Nm/kg); ABD_WL_: isometric hip abduction peak force for the weakest leg (N/kg); ABD_SL_: isometric hip abduction peak force for the strongest leg (N/kg); DHI: Dizziness Handicap Inventory (unitless).

**Table 4:** Rehabilitation priorities for people with multiple sclerosis according to the EDSS stage.

	Main Target	Complementary Activities
**MS1**	Balance exercises Hip strength exercises	Lower limb resistance training Core stability
**MS2**	Comprehensive program. Lower limb resistance training Balance and core stability exercises	Knee flexors resistance exercises Increased work out on the weakest leg Functional mobility task
**MS3**	Knee flexors resistance exercises Increased work out on the weakest leg Balance exercises Gait and functional mobility tasks	Core stability exercises Hip strength exercises

MS1: multiple sclerosis people with minimal impairment (EDSS ≤ 1.5); MS2: multiple sclerosis people with mild impairment (2 ≤ EDSS ≤ 3.5); MS3: multiple sclerosis people with moderate impairment (4 ≤ EDSS ≤ 6). Recommendations provided in the “Main Target” section were based on the main deficits observed in each multiple sclerosis group analyzed in this study. Recommendations provided in the “Complementary Activities” section were carried out to avoid the deficits that will probably occur in the next stage of the multiple sclerosis disease.
